# Bilayer Scaffolds of PLLA/PCL/CAB Ternary Blend Films and Curcumin-Incorporated PLGA Electrospun Nanofibers: The Effects of Polymer Compositions and Solvents on Morphology and Molecular Interactions

**DOI:** 10.3390/polym16121679

**Published:** 2024-06-13

**Authors:** Areeya Tuanchai, Phakanan Iamphring, Pattaraporn Suttaphakdee, Medta Boupan, Jaroslav Mikule, Juan Pablo Pérez Aguilera, Patnarin Worajittiphon, Yujia Liu, Gareth Michael Ross, Stepan Kunc, Petr Mikeš, Masafumi Unno, Sukunya Ross

**Affiliations:** 1 Biopolymer Group, Department of Chemistry, Center of Excellence in Biomaterials, Faculty of Science, Naresuan University, Phitsanulok 65000, Thailand; areeya08002@gmail.com (A.T.); phakanani63@nu.ac.th (P.I.); pattaraporn.su@nu.ac.th (P.S.); boupan.me@gmail.com (M.B.); gareth@nu.ac.th (G.M.R.); 2 Department of Chemistry, Faculty of Science, Humanities and Education, Technical University of Liberec, Studentská 1402/2, 461 17 Liberec, Czech Republic; jaroslav.mikule@tul.cz (J.M.);; 3 Department of Chemistry, Center of Excellence in Materials Science and Technology, Faculty of Science, Chiang Mai University, Chiang Mai 50200, Thailand; patnarin.w@cmu.ac.th; 4 Department of Chemistry and Chemical Biology, Faculty of Science and Technology, Gunma University, Tenjin-cho, Kiryu 376-8515, Japan; yliu@gunma-u.ac.jp (Y.L.); unno@gunma-u.ac.jp (M.U.); 5 Department of Physics, Faculty of Science, Humanities and Education, Technical University of Liberec, Studentská 1402/2, 461 17 Liberec, Czech Republic; stepan.kunc@tul.cz (S.K.); petr.mikes@tul.cz (P.M.)

**Keywords:** tissue engineering scaffolds, polylactide, curcumin, electrospun nanofibers, biodegradable

## Abstract

Tissue engineering scaffolds have been dedicated to regenerating damaged tissue by serving as host biomaterials for cell adhesion, growth, differentiation, and proliferation to develop new tissue. In this work, the design and fabrication of a biodegradable bilayer scaffold consisting of a ternary PLLA/PCL/CAB blend film layer and a PLGA/curcumin (CC) electrospun fiber layer were studied and discussed in terms of surface morphology, tensile mechanical properties, and molecular interactions. Three different compositions of PLLA/PCL/CAB—60/15/25 (TBF1), 75/10/15 (TBF2), and 85/5/10 (TBF3)—were fabricated using the solvent casting method. The electrospun fibers of PLGA/CC were fabricated using chloroform (CF) and dimethylformamide (DMF) co-solvents in 50:50 and 60:40 volume ratios. Spherical patterns of varying sizes were observed on the surfaces of all blend films—TBF1 (17–21 µm) > TBF2 (5–9 µm) > TBF3 (1–5 µm)—caused by heterogeneous surfaces inducing bubble nucleation. The TBF1, TBF2, and TBF3 films showed tensile elongation at break values of approximately 170%, 94%, and 43%, respectively. The PLGA/CC electrospun fibers fabricated using 50:50 CF:DMF had diameters ranging from 100 to 400 nm, which were larger than those of the PLGA fibers (50–200 nm). In contrast, the PLGA/CC electrospun fibers fabricated using 60:40 CF:DMF had diameters mostly ranging from 200 to 700 nm, which were larger than those of PLGA fibers (200–500 nm). Molecular interactions via hydrogen bonding were observed between PLGA and CC. The surface morphology of the bilayer scaffold demonstrated adhesion between these two solid surfaces resembling “thread stitches” promoted by hydrophobic interactions, hydrogen bonding, and surface roughness.

## 1. Introduction

The design and fabrication of polymeric materials have been thoroughly explored in the biomedical field, particularly for drug delivery applications. These materials have been developed in a variety of forms, including polymeric nanoparticles, polymeric micelles, mesoporous nanoparticles, dendrimers, hydrogels, polymeric nanofibers, and thin films [1]. Among these materials, polymeric scaffolds used in tissue engineering have gained significant attention due to their versatile performance in treatment. They meet the rising demand for human tissues and address the growing market need for tissue repairs resulting from acute illnesses [2,3,4]. However, ideal polymeric scaffolds for tissue engineering have yet to be developed. Their performance depends on a range of factors, including physical and mechanical properties, scaffold architecture, biodegradability, biocompatibility, cellular behavior, and the complex system required for self-healing within the human body [2].

The literature presents various tissue engineering polymeric scaffolds that are designed to mimic the mechanical properties and architecture of tissues, supporting cells as an extracellular matrix (ECM). Key aspects include porosity, pore diameter, pore structures, and surface-to-volume ratio [5,6]. Various scaffold types can be fabricated using diverse techniques like solvent casting, freeze-drying, electrospinning, phase separation, 3D printing, and bioprinting [7]. These methods utilize a range of natural and synthetic polymers [8,9,10]. Research indicates that new tissue engineering scaffolds with multiple layers can better mimic the complex biological environment of tissues compared to conventional monolayer scaffolds. This is because multi-layered scaffolds more closely resemble the natural multi-layered structure of biological tissues [11,12,13,14]. Therefore, the current study focuses on designing and fabricating bilayer polymeric scaffolds composed of a film layer and an electrospun layer. Two fabrication methods, solvent casting and electrospinning, were employed using biodegradable and biocompatible materials, both natural and synthetic polymers. 

Films and electrospun fibers, commonly used as tissue engineering scaffolds, are predominantly made from biodegradable and biocompatible polyesters approved by the FDA. These include poly(lactic acid) or polylactide (PLA), polycaprolactone (PCL), poly(glycolic acid) or polyglycolide (PGA), and their copolymers like poly(glycolide-co-caprolactone) (PGCL) and poly(lactide-co-glycolide) (PLGA) [15,16,17,18,19]. To further enhance these scaffolds, natural active materials, such as chitosan, silk protein, aloe vera, curcumin, honey, and cellulose, have been incorporated into their structures. Such incorporations improve their capabilities, including drug delivery and antibacterial properties [20,21,22]. Curcumin, a yellow active compound predominantly found in turmeric, is characterized by its phenolic structure. It holds great promise as a herbal drug due to its diverse therapeutic effects, including antioxidant, antimicrobial, and anti-inflammatory activities [23]. Curcumin is also recognized as an effective agent for drug delivery and wound dressings [24,25]. Therefore, curcumin (abbreviated as CC) was a suitable natural active material for this study. 

**Figure 1:**
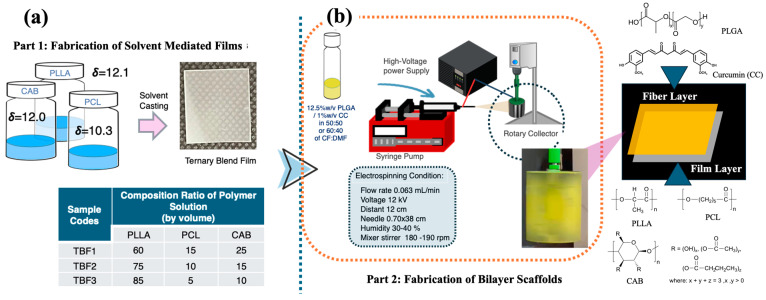
Schematic representation of this work: (**a**) fabrication of ternary blend films of PLLA/PCL/CAB using solvent casting and (**b**) fabrication of biodegradable bilayer scaffolds with a PLGA/CC electrospun layer and a PLLA/PCL/CAB ternary blend film layer, showing compositions for solvent casting, parameters for electrospinning, and chemical structures of all materials used.

In this study, a ternary blend film layer consisting of poly(L-lactide) (PLLA), polycaprolactone (PCL), and cellulose acetate butyrate (CAB) was fabricated using the solvent casting method. Different compositions of PLLA, PCL, and CAB, along with the effects of chloroform (CF) and dimethylformamide (DMF) co-solvents, were examined in terms of surface morphology, molecular interactions, and tensile mechanical properties (Figure 1a). The selection of PLLA, PCL, and CAB was based on the known miscibility between the polymer pairs PLLA/CAB and PCL/CAB. CAB enhances the compatibility of these blends, resulting in films with high tensile elongation, as observed in previous research [18,26]. An electrospun fiber layer, incorporating CC into PLGA, was also fabricated. The effect of the co-solvent system on the surface morphologies was studied, including that of pure PLGA. PLGA (a semi-crystalline polyester) was chosen due to its sufficient mechanical properties and biodegradation [15,16,27]. Finally, the most suitable ternary blend film and PLGA/CC electrospun fibers were selected to create a biodegradable bilayer scaffold. This was achieved by electrospinning the PLGA/CC solution onto the surface of the ternary blend films (Figure 1b). The surface morphology and adhesion between the film and electrospun fibers were analyzed using field emission scanning electron microscopy (FE-SEM). This newly designed bilayer scaffold, comprising a PLLA/PCL/CAB film layer and PLGA/CC electrospun fibers, is expected to provide strong mechanical support due to the film layer while offering a highly porous structure. The incorporation of curcumin adds active bioactivities, making the scaffold suitable for efficient tissue regeneration and drug delivery.

## 2. Experimental Section

### 2.1. Materials

Poly(lactide-co-glycolide) (PLGA), with an LA:GA ratio of 75:25 and an average molecular weight of 108,000 g/mol, and poly(L-lactide) (PLLA), with an average molecular weight of 190,000 g/mol, were supplied by the bioplastic production laboratory for medical applications, Faculty of Science, Chiang Mai University. Polycaprolactone (PCL) (Mn = 80,000 g/mol) was purchased from Shenzhen Esun Industrial Co., Ltd, Shenzhen, China. Cellulose acetate butyrate (CAB) (Mn = 30,000 gmol^−1^) was purchased from Sigma-Aldrich, Dorset, UK. Curcumin (CC) was purchased from MySkinRecipes, Bangkok, Thailand. Chloroform (CF) and dimethylformamide (DMF), used as solvents for electrospinning, were supplied by RCI Labscan Limited, Bangkok, Thailand.

### 2.2. Fabrication of Ternary Blends of PLA/PCL/CAB by Solvent Casting

Three different ternary blends of PLLA/PCL/CAB with compositions of 60/15/25, 75/10/15, and 85/5/10 were chosen to fabricate films using the solvent casting film method (see Figure 1a). Chloroform was used as a solvent. The rationale for the selection of these compositions derived from the results on the percentage of elongation (%elongation) of PLA/PCL/CAB film from our previous work on PLA/PCL/CAB melt-blown films. These compositions promoted high %elongation values in the range of 350–400%, which were much higher than those of pure PLA (approximately 20% elongation). In addition, the selection of biodegradable and biocompatible PLA, PCL, and CAB was studied and reported previously, where the partial miscibility between binary blends of PLA/CAB and PCL/CAB within ternary blends was calculated and predicted using critical solubility parameters—a method different from Coleman and Painter’s approach based on the difference in solubility values (δ) [26]. Briefly, 7 wt% solutions of individual polymers (PLA, PCL, and CAB) were mixed in chloroform in a total volume of 10 mL before fabrication in a polypropylene container. The mixture solution was then left for gradual solvent evaporation for 24 h in an acrylic container at room temperature (approximately 25 ± 4 °C) at 35–45% humidity to form films. All film samples were further dried in a vacuum at 40 °C for 24 h to remove residual solvents, producing films with a thickness of approximately 200 μm. 

### 2.3. Fabrication of Electrospun Nanofibers of PLGA: Effects of Co-Solvents

An electrospinning technique was used to produce nanofibers that have large surface areas and porous structures which can be designed to be similar to the extracellular matrix (ECM) to enhance cell adhesion, proliferation, and migration [21]. Two different co-solvents of chloroform (CF) and dimethylformamide (DMF) at 50:50 and 60:40 were chosen for use as solvent systems for the electrospinning of PLGA and curcumin (CC)-incorporated PLGA. PLGA at 12.5% *w*/*v* was used and dissolved in the co-solvents (50:50 CF:DMF and 60:40 CF:DMF). The solutions were mixed until homogeneity for 24 h before being loaded into a 10 mL syringe for electrospinning. Electrospinning was conducted with our own customized setup composed of a syringe pump, a 22G × 1½-inch blunt needle (0.7 × 40 mm thin-wall hypodermic needle), a Genvolt 73030 variable high-voltage DC power supply, and a rotary drum for sample collection. The electrospun samples were prepared at a flow rate of 0.063 µL/min, with 12 kV output from the power supply, a distance of 12 cm between the nozzle tip and the grounded collector, and controlled humidity of approximately 30–45%. A rotating drum (175 rpm) was used to collect the spun nanofibers. During the electrospinning process, the PLGA solution was injected from the spinneret needle tip to give a pendant droplet generated from the surface tension. Once an electric field was applied, electrostatic repulsion among the like-charged surface particles deformed the polymer droplet into a Taylor cone, from which a charged jet was ejected and then formed fibers. The fabricated fibers were vacuumed to remove any residual solvent before being stored in a dry atmosphere before characterization.

### 2.4. Fabrication of Electrospun Nanofibers of PLGA Incorporated with Curcumin (CC)

The mixture solution of 12.5% *w*/*v* PLGA (either 50:50 or 60:40 CF:DMF) incorporated with dried powder of curcumin (CC) at 1.0% wt of the total weight of the PLGA was prepared. The solutions were mixed until homogeneity for 24 h before being loaded into a 10 mL syringe. Electrospinning was conducted with the same conditions used for the electrospinning of PLGA. The fabricated fibers were vacuumed to get rid of any residue solvent before being stored in a dried atmosphere before the characterizations and the comparison of their results with those of the PLGA fibers.

### 2.5. Fabrication of Biodegradable Bilayer Scaffolds

Biodegradable bilayer scaffolds composed of electrospun fibers and films were designed and fabricated. The ternary blend film of PLLA/PCL/CAB with the optimal composition was selected and fabricated into a film (following the above method) before being attached to the rotary drum used as a collector. The polymer solutions of either PLGA or PLGA/CC at optimal co-solvent ratios were prepared and used to fabricate electrospun fibers. The solutions were spun onto the film surface tapped at the collector (see Figure 1b). The bilayer scaffolds of the PLGA/CC electrospun nanofibers and the ternary blend film of PLLA/PCL/CAB were produced and characterized for their surface morphologies.

### 2.6. Characterizations

#### 2.6.1. Functional Groups Determined by FT-IR

The functional groups of the PLLA/PCL/CAB ternary blend films, electrospun fibers of PLGA, and electrospun fibers of PLGA/CC were determined with the Fourier transform infrared spectrometry (FT-IR) technique using a Perkin Elmer model, the Spectrum GX. Briefly, the samples were dried at 50 °C for 60 min before being tested using the attenuated total reflection (ATR) mode of FTIR with the spectrometer at 4000–400 cm^−1^.

#### 2.6.2. Surface Morphology Determined by FE-SEM

The morphologies of the ternary blend films of PLLA/PCL/CAB, the electrospun fibers of PLGA, and the electrospun fibers of PLGA/CC were observed using field emission scanning electron microscopy (FE-SEM, mode STEM) (Apreo S, Thermo Fisher Scientific, Waltham, MA, USA). All samples were prepared and tapped onto grids before being sputtered with gold particles before the examination of their surface morphologies using different magnifications between 500× and 10,000×.

#### 2.6.3. Fiber Diameter Size Measurement

The SEM images of the electrospun fibers were used to determine the diameter sizes of the fibers, and the size distribution of the fibers was reported. The size distribution was analyzed using ImageJ software (FIJI 1.46 version I.J 1.46r by SciMark 2.0).

#### 2.6.4. Tensile Properties

The ternary blend films of PLLA/PCL/CAB with different compositions were tested for their tensile strength following ASTM D638. All samples were measured with a universal tensile machine (UTM; INSTRON^®^ CALIBRATION LAB, Model 5965, Hopkinton, MA, USA) at 25 °C and 40% humidity. The tensile testing was carried out using a 1 KN load cell and an extension rate of 20 mm/min. The average of five measurements for each test was calculated for the samples.

## 3. Results and Discussion

The design and fabrication of bilayer scaffolds consisting of films and electrospun fibers were studied. In the initial phase, ternary blend films of PLLA/PCL/CAB were produced using solvent casting. Their morphology, molecular interactions, and mechanical properties were analyzed to assess the impact of different polymer compositions. In the second phase, electrospun nanofibers of PLGA and PLGA incorporated with CC were fabricated while studying the effect of the solvents used during electrospinning. The fabrication of the bilayer structure, consisting of the films and electrospun fibers, was completed, and the morphology of the layers was carefully examined.

### 3.1. Ternary Blend Films: Effects of Polymer Compositions

Ternary blend films of PLLA/PCL/CAB were fabricated with three different compositions—60/15/25, 75/10/15, and 85/5/10—that were selected from our previous work [26]. Films with these compositions demonstrated high elongation (exceeding 350%) using the in situ compatibilized melt-blown film technique with PLA/PCL/CAB. Generally, the rationale behind the fabrication technique is crucial for determining a film’s physical and mechanical properties, especially when comparing melt-blending and solvent-blending methods. In this study, the solvent-mediated blending method was utilized to improve the dispersion of the three polymer phases, PLLA, PCL, and CAB, in the specified compositions. It is important to note that PLLA, a semi-crystalline polymer, has a laboratory-grade L-lactide stereochemical structure. In contrast, PLA, of melt-processing grade for commercial-scale production, includes additional fillers to make it suitable for melt processing and final applications.

#### 3.1.1. Surface Morphology

**Figure 2:**
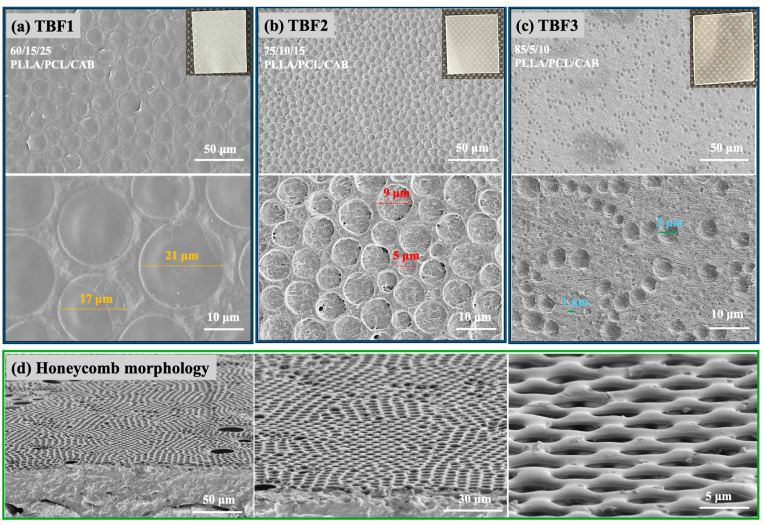
SEM images of ternary blend films of PLLA/PCL/CAB with different compositions showing their spherical morphology at magnifications of 500× (50 µm scale bar) and 2000× (10 µm scale bar) (**a**–**c**) together with optical film images and SEM images of the ternary blend film with 60/15/25 PLLA/PCL/CAB (at different processing times for TBF1) showing a honeycomb morphology at magnifications of 500×, 1000×, and 5000× (**d**).

The surface morphology of all the solvent-mediated blend films (TBF1, TBF2, and TBF3) (Figure 2a–c) exhibited spherical patterns. The spherical structures in TBF1 (17–21 µm) were larger than those in TBF2 (5–9 µm) and TBF3 (1–5 µm). This size variation is attributed to the different amounts of polymers in the blend films, with higher amounts of PLLA and lower amounts of PCL and CAB. This phenomenon is complex, but a possible explanation involves the ordered crystallized PLLA (the film matrix), the flexible amorphous and crystalline chains of PCL, and the amorphous hydrogen-bonded CAB. These factors collectively create heterogeneous surfaces that induce bubble nucleation [28]. The fewer crystalline structures of PLLA and the more flexible chains of PCL in the polymer solution allowed CAB chains to intermingle, promoting larger phases. Additionally, the higher adsorption of water molecules in TBF1, which occurred in the open-air system during the solvent casting technique, can be attributed to the greater amount of CAB. Despite monitoring temperature and humidity, these conditions were not perfectly controlled. This led to molecular interactions with PLLA, PCL, and water through hydrogen bonding [18,26], creating a higher supersaturation condition conducive to bubble nucleation. 

**Figure 3:**
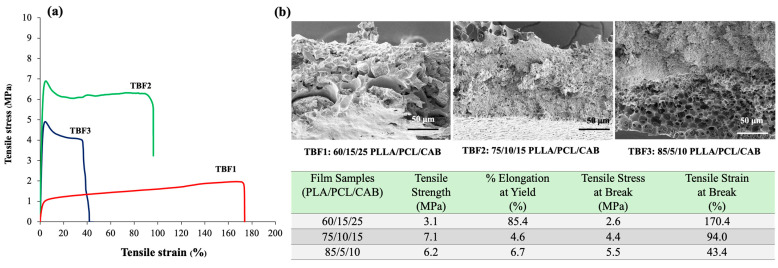
Tensile curves (**a**) and tensile fractured-surface morphologies (**b**) of ternary blend films of PLLA/PCL/CAB with different compositions.

In addition, honeycomb structures were unexpectedly and unusually observed on the surface of the ternary blend of PLLA/PCL/CAB at a ratio of 60/15/25 with different manufacturing times for the solvent casting of TBF1 (Figure 2d). These honeycomb micrometer-scaled polymer patterns were caused by the condensation of water droplets, which occurs when water vapor contacts a cold surface. This phenomenon is associated with the rapid evaporation of polymer solutions exposed to humidity [29,30,31]. The mechanism behind the formation of this honeycomb morphology is complex, involving thermodynamics and transport phenomena. A possible explanation is that the rapid evaporation of the solvent increases the superficial concentration, causing cooling of the solution surface and subsequently leading to the condensation of water. This process leads to phase separation with the formation of a polymer film at the solution surface, possibly around water droplets. These droplets create holes in the superficial spheres, facilitating the evaporation of both the solvent and water [32,33]. Therefore, the PLLA/PCL/CAB blend with a composition of 60/15/25 exhibited a microporous pattern due to various parameters: specific phase separation and agglomeration of PLLA, PCL, and CAB; evaporation of the polymer solution; nucleation, condensation, and growth of water droplets; evaporation of water and solvent; and solidification of the polymer [34]. These explanations can also describe the formation of porous structures in TBF1, TBF2, and TBF3, which can be observed in the cross-sectional FE-SEM images from the tensile test shown in Figure 3.

#### 3.1.2. Tensile Properties

Three different solvent-mediated ternary blend films of PLLA/PCL/CAB were tested for their tensile strength and elongation (Figure 3). The TBF1 film exhibited the highest tensile elongation at break, approximately 170%, followed by TBF2 at 94% and TBF3 at 43%. In contrast, the tensile strength of TBF2 was higher than that of both TBF1 and TBF3. In addition, the TBF1 film was softer and tougher than TBF2 and TBF3, respectively, when they were physically toughed by hand. In contrast, TBF3 was more brittle than TBF2 and TBF1. This reveals that the polymer compositions of PLLA, PCL, and CAB affected the tensile mechanical properties of the solvent-mediated ternary blend films, in which high amounts of PLLA resulted in more brittle films, consistent with the findings from previous reports on melt-blown films [26]. The different deformation and disintegration behaviors of these films may be attributed to the distinct formation of PLLA, PCL, and CAB phases, which influence the viscosity, elasticity ratio, and interfacial tension during solvent evaporation. These factors correlate with the observed surface morphology and the formation of porous structures in the films described earlier. All blend films showed higher tensile elongation than pure PLLA film, which has a %elongation of less than 10%. 

#### 3.1.3. Molecular Interactions Observed by FT-IR

**Figure 4:**
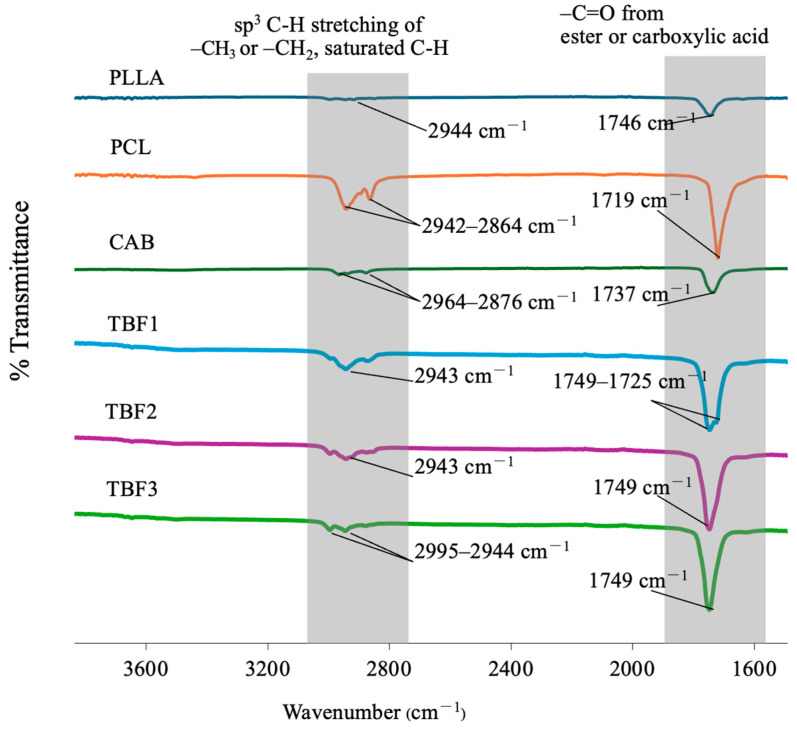
FT−IR spectra of ternary solvent-mediated blends of PLLA/PCL/CAB with different compositions of PLLA, PCL, and CAB.

After observing the tensile strength and elongation of all the ternary blend films (TBF1, TBF2, and TBF3), which were significantly higher than those of pure PLLA, the molecular interactions of these blends were further studied and discussed in terms of miscibility, as observed by the FT-IR technique. This analysis is crucial because the mechanical performance of ternary polymer blends is influenced by their miscibility, which depends on the competition between recrystallization and intermixing of the polymers [18,35,36,37]. Figure 4 shows the FT-IR spectra of PLLA, PCL, and CAB and their blends in TBF1, TBF2, and TBF3. PLLA, PCL, and CAB all exhibit absorption bands corresponding to -C-H stretching of saturated -CH, -CH_2_, and -CH_3_ at 2750–3000 cm^−1^ and absorption bands of -C=O stretching from ester or carboxylic acid groups at 1700–1760 cm^−1^. A shift and broadening of the FT-IR absorption bands of carbonyl groups were observed in all ternary blends (TBF1, TBF2, and TBF3) compared to pure PLLA, PCL, and CAB. This can be caused by diminished intermolecular packing of PLLA, PCL, and CAB chains, along with potential molecular interactions, such as hydrogen bonding between CAB/PLLA or CAB/PCL. In these circumstances, the electron cloud distribution around carbonyl groups is changed, consequently affecting the resonant frequency and breadth of bands [38]. It is important to note that distinguishing the differences in the molecular interactions among the three ternary blend compositions (TBF1, TBF2, and TBF3) was challenging. However, the results from FT-IR demonstrated that all ternary solvent-mediated blends of PLLA/PCL/CAB at 60/15/25, 75/10/15, and 85/5/10 were most likely to be miscible or partially miscible, resulting in an enhancement of tensile elongation when compared to that of pure PLLA.

### 3.2. PLGA Electrospun Fibers: Effects of Co-Solvents and Curcumin on the Morphology and Molecular Interactions

Poly(lactide-co-glycolide) (PLGA) with an LA:GA ratio of 75:25 was selected to fabricate nanofibers using the electrospinning technique. This polymer is biodegradable and biocompatible, approved by the Food and Drug Administration (FDA), and mechanically robust, making it suitable for use in medical applications, particularly in drug delivery systems. Generally, the solvent used for electrospinning is a crucial parameter in the formation of fibers. In this study, chloroform (CF) and N,N-dimethylformamide (DMF) co-solvents were used at different CF:DMF ratios of 50:50 and 60:40 to dissolve PLGA at a consistent concentration of 12.5% *w*/*v*. The effects of these solvent ratios on the morphology of PLGA fibers were then studied. Additionally, 1% wt of curcumin (CC), a natural active agent, was incorporated into the PLGA solution and fabricated into fibers. The effects of the solvent ratios on their surface morphology and molecular interactions were studied. It is important to note that all other parameters, including %humidity, polymer concentration, spinning flow rate, the distance between the tip and the collector, and applied voltage, were controlled throughout this study.

#### 3.2.1. Morphology of PLGA Electrospun Fibers: Effects of Co-Solvents

**Figure 5:**
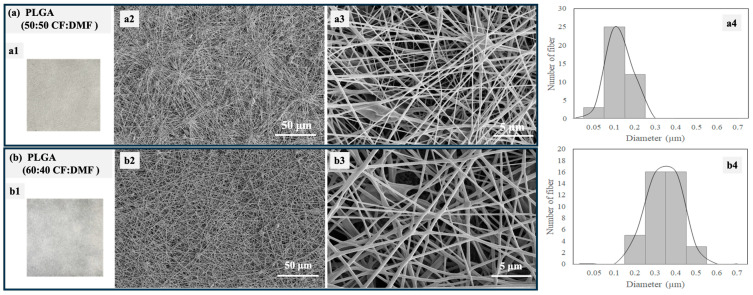
Optical photographs (**a1**,**b1**), SEM images (with different magnifications, **a2**,**a3**,**b2**,**b3**), and size distributions of PLGA electrospun fibers fabricated using different ratios of solvents (**a4**,**b4**): (**a**) 50:50 CF:DMF and (**b**) 60:40 CF:DMF.

Figure 5 shows optical photographs, SEM images, and size distributions of PLGA electrospun fibers fabricated using different ratios of the co-solvents CF and DMF. The optical photographs of the fibers are similar, as expected (Figure 5(a1,b1)). Smooth and well-defined PLGA fibers were successfully fabricated with a small number of beads. However, different fiber diameters were observed depending on the solvent ratios. The PLGA fibers had diameters ranging from 50 to 200 nm when a 50:50 CF:DMF ratio was used (Figure 5(a2–a4)). In contrast, larger fiber diameters, ranging from 200 to 500 nm, were observed with a 60:40 CF:DMF ratio (Figure 5(b2–b4)). In our previous work, we reported that the diameter sizes of electrospun fibers of PLGA were 1000–2000 nm when 80:20 CF:DMF was used [27]. 

The higher proportion of CF in the co-solvent led to larger diameter sizes of the PLGA electrospun fibers due to its high evaporation rate, which is related to its low boiling point (approximately 60 °C). In contrast, DMF, with its high boiling point (approximately 153 °C), is not as easily volatilized during the electrospinning process. The strong dielectric ability of the polymer jet containing a high proportion of DMF generated a large electrostatic drafting force in the stable electrospinning, resulting in the formation of smaller-diameter PLGA fibers [39,40]. In addition, a larger number of beads was observed in PLGA fibers using the 50:50 CF:DMF ratio [Figure 5(a2)] compared to fibers using the 60:40 CF:DMF ratio [Figure 5(b2)]. Generally, bead formation is due to insufficient entanglement of polymer molecular chains, often caused by low polymer concentrations. In this study, the PLGA concentration was maintained at 12.5% *w*/*v*. Therefore, the different proportions of these co-solvents might have had a minor effect on the polymer concentration during spinning, primarily due to the differing evaporation rates of CF and DMF [41,42].

#### 3.2.2. Morphology of PLGA/CC Electrospun Fibers

**Figure 6:**
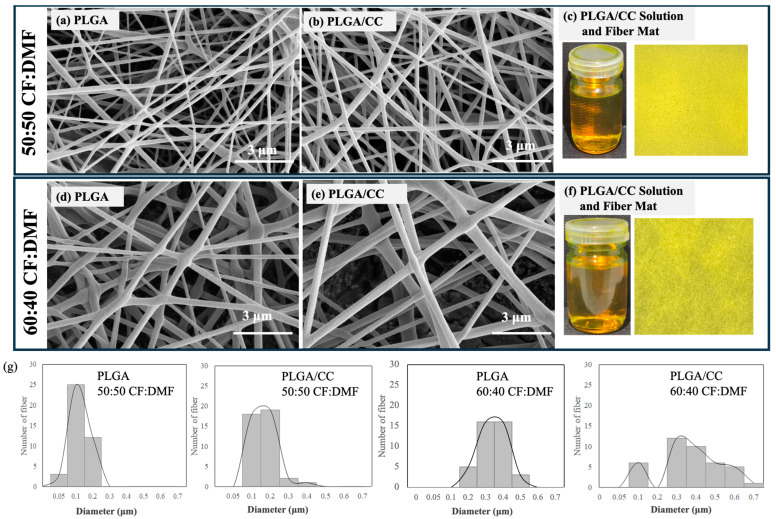
SEM images of PLGA electrospun fibers fabricated using 50:50 CF:DMF (**a**) and 60:40 CF:DMF (**d**), SEM images of PLGA/CC electrospun fibers fabricated using 50:50 CF:DMF (**b**) and 60:40 CF:DMF (**e**), Optical photographs of PLGA/CC solution dissolved in 50:50 CF:DMF and its electrospun fiber mat (**c**), Optical photographs of PLGA/CC solution dissolved in 60:40 CF:DMF and its electrospun fiber mat (**f**), along with their diameter size distributions (**g**).

The electrospun fiber layer was further designed to incorporate curcumin (CC) (1% wt) with PLGA (12.5% *w*/*v*) in co-solvent systems of 50:50 and 60:40 CF:DMF. CC, an active ingredient of turmeric and a natural polyphenolic compound, was chosen due to its promising therapeutic properties [1,19,22]. CC is a yellow-colored phytochemical that is hydrophobic and frequently soluble in organic solvents such as dimethylsulfoxide, ethanol, and acetone, as well as DMF and CF. The morphology and diameter sizes of the PLGA/CC electrospun fibers were compared with those of the PLGA electrospun fibers (Figure 6).

As can be seen in Figure 6, the electrospun fiber mats of PLGA/CC appeared bright yellow due to the addition of CC, while the PLGA mat appeared white. The PLGA/CC fibers exhibited a smooth surface with a uniform structure in both co-solvent systems of 50:50 and 60:40 CF:DMF. The PLGA/CC electrospun fibers fabricated using 50:50 CF:DMF had diameters ranging from 100 to 400 nm—larger than the 50–200 nm range of PLGA fibers. Similarly, the PLGA/CC electrospun fibers fabricated using 60:40 CF:DMF had diameters mostly in the range of 200–700 nm—larger than the 200–500 nm range of PLGA fibers. This indicates that a higher proportion of CF in the co-solvent promotes larger diameter sizes in PLGA/CC fibers, consistent with the results observed for PLGA fibers. This is due to the effect of solvent evaporation, as discussed earlier. Additionally, the incorporation of CC into PLGA increased the diameter sizes of the electrospun fibers, likely due to the higher concentration in the PLGA/CC solution compared to the PLGA solution. A higher polymer concentration in the electrospinning solution results in increased viscosity and surface tension. The solution viscosity influences the movement and entanglement of polymer chains within the spinning solution [41,42]. This enables the solvent molecules to be distributed over the entangled polymer chains, promoting a viscoelastic force that limits the stretching effect of the electrostatic and Coulombic repulsion forces. As a result, this leads to an increase in fiber diameter [43,44,45]. 

#### 3.2.3. Chemical Functional Groups of PLGA/CC Electrospun Fibers

**Figure 7:**
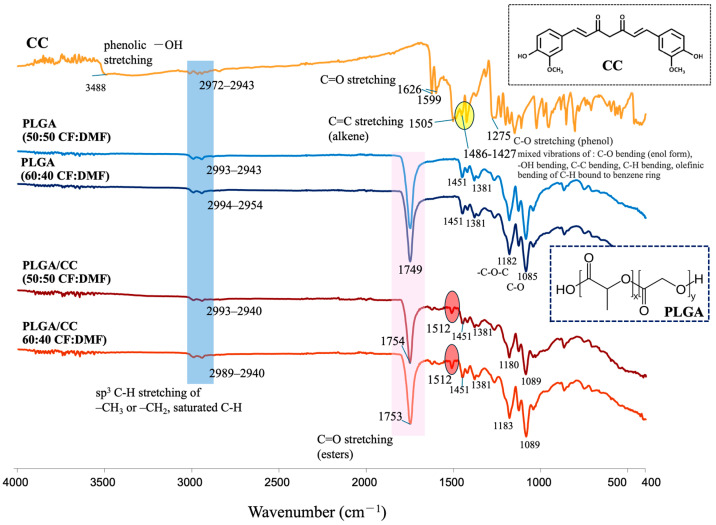
FT-IR spectra of CC, PLGA, and PLGA/CC electrospun fibers fabricated using co-solvent ratios of 50:50 and 60:40 CF:DMF.

The chemical functional groups of CC, PLGA, and PLGA/CC fabricated in the co-solvent were also analyzed (Figure 7). The characteristic FTIR spectrum peaks of CC showed phenolic -OH stretching vibration at 3488 cm^−1^, -C=O stretching at 1626 cm^−1^ and 1599 cm^−1^, stretching vibration of C=C of alkene at 1505 cm^−1^, and mixed vibrations of C-O bending of enol form, O-H and C-C bending, and olefinic bending of C-H bound to benzene rings at 1486–1427 cm^−1^ [46,47,48], whereas PLGA (spun using both 50:50 and 60:40 CF:DMF) showed mainly -C=O stretching at 1749 cm^−1^ and C-O-C and C-O vibrations at 1182 and 1085 cm^−1^, respectively. The FTIR spectra of the PLGA/CC electrospun fibers (spun using both 50:50 and 60:40 CF:DMF) showed the characteristic peaks of both CC and PLGA. However, the absence of phenolic -OH stretching, a small shift in -C=O stretching (ester), and the formation of stronger peaks observed at 1512 cm⁻¹ indicate that PLGA and CC bonded together. This bonding was likely due to interactions between the carbonyl groups of PLGA and the phenolic hydroxyl groups of CC. This interaction was enhanced by the improved dispersion of CC (a hydrophobic filler) into PLGA (a hydrophobic material), resulting in a strong bond between the two materials [49].

### 3.3. Biodegradable Bilayer Scaffolds

**Figure 8:**
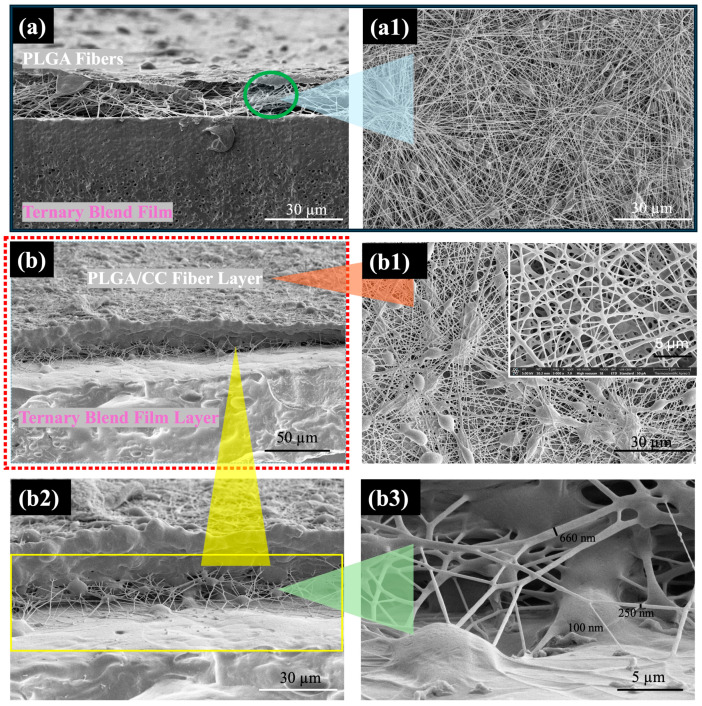
SEM images of bilayer scaffolds of PLGA electrospun fibers and the ternary blend film of PLLA/PCL/CAB ((**a**) side-view demonstrating the PLGA electrospun fibers on top of the film (**a1**)) and of PLGA/CC electrospun fibers and the ternary blend film of PLLA/PCL/CAB ((**b**) side-view demonstrating the PLGA/CC electrospun fiber (**b1**)) showing the area of the adhesion between the fibers and the film at different magnifications (**b2**,**b3**).

The biodegradable bilayer scaffolds were designed and fabricated by electrospinning PLGA/CC (in a 50:50 CF:DMF co-solvent) onto a ternary blend film of 75/10/15 PLLA/PCL/CAB. The 50:50 CF:DMF ratio was chosen for its ability to produce nanoscale fibers, while the 75/10/15 PLLA/PCL/CAB composition was selected for its sufficient strength and elongation. The morphologies of the bilayer scaffolds, consisting of either PLGA or PLGA/CC (as the fiber layer) and the ternary blend film of PLLA/PCL/CAB (as the film layer), were observed (Figure 8). The two distinct layers of PLGA and the film (Figure 8a), as well as PLGA/CC and the film (Figure 8b), were clearly visible. Interestingly, the adhesion between the two different solid surfaces (fibers and film) was clearly noticeable, resembling “thread stitches” of PLGA/CC fibers on the film surface, with fiber diameters ranging from 100 to 600 nm. The bilayer scaffolds adhered firmly together, making them difficult to peel apart manually. Additionally, it was observed that the PLGA/CC fibers adhered on the film surface stronger than the PLGA fibers when peel strength was assessed by hand. These results suggest strong interfacial adhesion between the hydrophobic PLGA/CC fibers and the hydrophobic PLLA/PCL/CAB film. Typically, two different solid materials must share similar properties, such as hydrophilic fillers with hydrophilic matrices or hydrophobic fillers with hydrophobic matrices, to create a strong bond between them [49]. 

**Figure 9:**
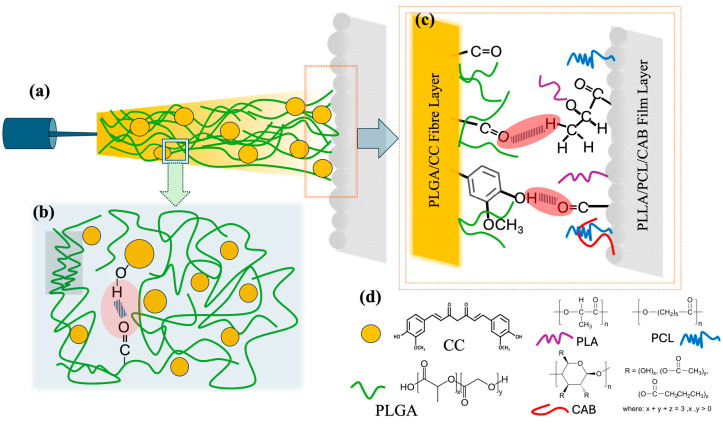
Schematic interaction bonding of PLGA/CC electrospun fibers on the ternary blend film of PLA/PCL/CAB; electrospun fiber jet contained PLGA molecular chains and curcumin (**a**), molecular interaction between hydroxyl group of CC and carbonyl group of PLGA (**b**), molecular interaction between two layers of PLGA/CC fiber layer and PLLA/PCLCAB film layer (**c**), and chemical structures and representative symbols of PLGA, CC, PLLA, PCL and CAB (**d**).

Figure 9 illustrates the interfacial adhesion between the PLGA/CC fibers and the PLLA/PCL/CAB film. When the surfaces of the PLGA/CC fibers and the PLLA/PCL/CAB film come into close proximity, atoms in one material begin to experience the electronic environment of the other, resulting in adhesive interaction. In this case, hydrophobic–hydrophobic molecular interfacial adhesion occurs due to the molecular orientation between the fiber and film surfaces. This interaction is further strengthened by some hydrogen bonding between molecules, such as the carbonyl groups of PLLA, PCL, and CAB and the hydroxyl groups of the phenolic-containing CC, or between the carbonyl groups of CC or PLGA and the hydrogen (methyl groups) in PLLA, PCL, and CAB. Additionally, the surface roughness of the PLLA/PCL/CAB film may enhance interfacial adhesion between the fibers and the film, further aided by humidity.

## 4. Conclusions

The design and fabrication of biodegradable bilayer scaffolds were successfully achieved using two different hydrophobic materials, electrospun fibers of PLGA/CC and a ternary blend film of PLLA/PCL/CAB, by studying the effects of polymer compositions and solvents on the properties of each layer, especially surface morphology and molecular interactions. The aim was to use a film (with sufficient mechanical properties and roughness) as a supporting layer for electrospun fibers to serve as an extracellular matrix for cell adhesion and proliferation). In the film fabrication, various compositions of PLLA, PCL, and CAB influenced the surface morphology, molecular interactions, and tensile mechanical properties of the film. The 75/10/15 composition demonstrated an optimal tensile strength and elongation percentage. For electrospun fiber fabrication, the CF and DMF co-solvent ratio significantly affected the fiber diameter of both PLGA and PLGA/CC, with a higher DMF promoting smaller fiber diameters (50–200 nm). Incorporating CC into PLGA resulted in larger fiber diameters (100–400 nm). Molecular interactions, particularly hydrogen bonding, were observed between PLGA and CC. Strong interfacial adhesion between the PLLA/PCL/CAB film and the PLGA/CC electrospun fibers was attributed to both hydrophobic interactions and hydrogen bonding, especially between the carbonyl and hydroxyl groups in their molecular structures. 

## Data Availability

The raw/processed data required to reproduce these findings cannot be shared at this time as the data form part of an ongoing study.
